# Eye Movement Desensitization and Reprocessing in adolescents with autism; Efficacy on ASD symptoms and stress

**DOI:** 10.3389/fpsyt.2023.981975

**Published:** 2023-02-15

**Authors:** Esther Marion Leuning, Iris van den Berk-Smeekens, Martine van Dongen-Boomsma, Wouter G. Staal

**Affiliations:** ^1^Karakter Child and Adolescent Psychiatry University Centre, Nijmegen, Netherlands; ^2^Donders Centre for Cognitive Neuroimaging, Donders Institute for Brain, Cognition and Behaviour, Radboud University, Nijmegen, Netherlands; ^3^Leiden Institute for Brain and Cognition, Leiden University, Leiden, Netherlands

**Keywords:** autism spectrum disorder, eye movement desensitization and reprocessing (EMDR), stress, adolescents, children

## Abstract

**Introduction:**

Eye Movement Desensitization and Reprocessing (EMDR) is a well-established and thoroughly researched treatment method for posttraumatic stress symptoms. When patients with an autism spectrum disorder (ASD) are treated with EMDR for their Posttraumatic Stress Disorder (PTSD), they sometimes report a decrease in the core symptoms of ASD. This explorative pre-post-follow up design study is designed to investigate whether EMDR with a focus on daily experienced stress, is effective in reducing ASD symptoms and stress in adolescents with ASD.

**Methods:**

Twenty-one adolescents with ASD (age 12 to 19) were treated with ten sessions EMDR, focusing on events of daily experienced stress.

**Results:**

No significant decrease of ASD symptoms was found on the total score of the Social Responsiveness Scale (SRS) as reported by caregivers from baseline to end measurement. However, there was a significant decrease on total caregivers SRS score comparing the baseline to the follow-up measurement. On two subscales, Social Awareness and Social Communication, a significant decrease was found from baseline to follow-up. On the subscales Social Motivation and Restricted Interests and Repetitive Behavior, no significant effects were found. On pre- and posttest scores of total ASD symptoms measured by the Autism Diagnostic Observation Schedule (ADOS-2), no significant effects were found. On the contrary, scores on self-reported Perceived Stress Scale (PSS) showed a significant decrease from baseline to follow-up. Also, 52% of adolescents showed a significant improvement of global clinical functioning at endpoint measurement on the Clinical Global Impression Improvement, rated by an independent child psychiatrist.

**Discussion:**

In sum, these results of this uncontrolled study suggest a partial effect of EMDR in adolescents with ASD on ASD symptoms, rated by their caregivers. In addition, the results of this study show that EMDR treatment on daily experienced stress significantly reduce perceived stress as reported by the participants, and improves global clinical functioning. The results also suggest a ‘sleeper effect’, since no significant effects were found between baseline- and post- treatment measurements, but only between baseline- and follow up three months after the treatment. This finding is in line with other studies investigating psychotherapeutic effects in ASD. Implications for clinical practice and suggestions for future research are discussed.

## Introduction

Autism spectrum disorder (ASD) is a common neurodevelopmental disorder with an average estimated prevalence of around 1% (1, 2).

For most individuals core ASD symptoms (i.e., deficits in communication, social-emotional reciprocity and restricted and repetitive behavior and interests) are present throughout life and place a great mental burden on individuals and their families (3, 4). Often, individuals with ASD report a lower quality of life (5), show impaired family-functioning due to for example elevated stress levels (6), a limited social network, and less meaningful relationships (7).

Adults with ASD perceive more stress in their daily life, and have less capacity to cope with this stress than mentally healthy individuals (8). This stress may cause lower social functioning. In addition, the stress itself may contribute to social disfunctioning of adults with ASD (9–11). Also, in childhood elevated stress levels may increase the risk of social communication impairments by triggering avoidance behavior, and than may further complicate development of the already hampering social skills (12–14). Focussing on reducing stress may be a new gateway to treatment options on social and emotional functioning in ASD. In general, psychotherapeutic interventions focussing on reducing stress in children and adolescents diagnosed with ASD, show positive effects. For instance, a meta-analysis on the effectiveness of mindfulness based therapies in reducing psychological distress in children with ASD and their caregivers, showed significant gains in subjective wellbeing and decrease of psychological distress, after treatment, and at three month follow-up. (15). Also, studies using a prepost design on the effects of Acceptance and Commitment Therapy (ACT) showed a reduction of stress, more pro-social behavior, more social awareness and more social cognition in children with ASD (8, 13, 16).

Among others, Eye Movement Desensitization and Reprocessing (EMDR) may be considered as a way to increase social skills by reducing stress in individuals with ASD. EMDR is a well-established treatment for adults and children with post-traumatic stress disorders (PTSD) (17–20). Different studies suggest that EMDR may be effective for several other conditions than PTSD; for instance in groups with extreme stress as a result of painful medical examination, spinal cord injury or extreme stress in pregnant women with a history of stillbirth (21). In these studies only a few EMDR sessions diminished perceived stress or anxiety.

One of the main hypothesized working mechanism of EMDR is that EMDR reduces the vividness of images of unpleasant memories held up in the working memory capacity by eye movements that concurrently use up processing resources in the working memory capacity (22–24). Working memory has been supposed to be an important fundamental higher-order function, underlying other executive functions (25) and is found to be impaired in individuals with ASD (26–29). A possible hypothesis for reducing core symptoms of ASD when treating an individual with ASD with EMDR, may be that stressful thoughts and images no longer occupy the already impaired working memory and so more mental space is left for social interactions.

First case studies in adults with ASD treated by EMDR for their PTSD complaints, reported significant progress in overall functioning (30, 31). Lobregt van Buuren et al. (32) showed in adults with ASD and a history of adverse events and trauma, that on one hand after 7 sessions EMDR a reduction was reported in core ASD symptoms, measured by the Social Responsiveness Scale-Adults and on the other hand a significant reduction of trauma-related symptoms and psychological distress. Furthermore, Leuning et al. (33) presented a case study in which an adolescent with ASD was treated with 10 weekly sessions of EMDRfor daily stress and confusion. Although measurements did not show a significant increase of social responsiveness or decrease in symptoms of ASD, a decrease was found in caregiver-reported restricted interest and repetitive behavior. In addition, caregivers reported better daily functioning in personal hygiene, improved school attendance and a greater ability to share feelings and thoughts. Comparing caregiver-based scores and self-reported scores on the SRS, the youngster in this case study reported no significant reduction in ASD symptoms. In research literature it is described that especially adolescents may have difficulties in properly reporting their own ASD symptoms (34).

In order to search for a novel entrance to improve social functioning in ASD, this explorative uncontrolled study was designed to investigate whether EMDR on daily experienced stress is effective in perceived stress, reducing ASD core symptoms and/or improving global functioning in adolescents with ASD.

## Materials and methods

### Participants

This study with a pre-post-follow up design was conducted at the outpatient care department of Karakter University Centre Nijmegen, an academic center for child and adolescent psychiatry in The Netherlands between November 2017 and July 2020. All adolescents were formally diagnosed with ASD by an experienced and multi-disciplinary team and classified by the criteria from DSM-IV-TR (35) or DSM 5 (3). Comorbid psychiatric disorders were allowed, except PTSD. Other inclusion criteria were a full-scale IQ of 80 or higher and the ability to understand and speak Dutch. Patients were excluded when they had PTSD complaints like flashbacks, reliving’s, nightmares and strong avoidance tendencies, reported in an interview based on the Clinician Administered PTSD Scale for Children and Adolescents (36). No other treatments were allowed during the study except pharmacotherapy with stable dosages of medication.

### Measures

#### Primary outcome

The total score of the Social Responsiveness Scale (SRS-A) (37) completed by one of the caregivers was used to assess the change in ASD symptoms prior, during, and after treatment. The SRS-A assesses the ability in adolescents to engage in reciprocal social behavior in natural social settings, measured in four subscales: (1) Social Awareness, (2) Social Communication (3) Social Motivation, and (4) Restricted Interests and Repetitive Behavior. In total, the four subscales comprise 65 items that are answered on a 4-point scale (never true to almost always true). The total score of the caregiver-rated SRS served as the primary outcome measure. Because of doubts in the research field as to whether young people are able to properly report their complaints themselves, the parent score was chosen as the primary outcome measure (34).

Cronbach’s alpha for SRS-A completed by caregivers was 0.95. Based on earlier research with a same research population on Pivotal Response Treatment (38, 39) a clinical responder on the SRS-A was defined as a reduction of > 25% on the total SRS score.

#### Secondary outcomes

The total score of SRS-A of the adolescents self-report was used as a secondary outcome measure. Also based on earlier research with a same research population on Pivotal Response Treatment (38, 39) a clinical response on the SRS-A was defined as a reduction of > 25% on the total SRS score.

Cronbach’s alpha for the SRS-A completed by adolescents, was 0.96. SRS-A total score had a sensitivity of 0.85 and a specificity of 0.83 for ASD versus typically developing participants. Correlations with established ASD scales were moderate to high (*r* = 0.25 −0.83) (40).

Another secondary outcome measure is the Autism Diagnostic Observation Schedule 2 (ADOS-2) (41), which is a semi-structured observation schedule in which the clinician elicits social, communicative, stereotyped and play behavior to observe symptoms of ASD. Observations of the clinician, who is blinded for baseline outcomes, are categorized and a score is assigned for each domain of ASD symptoms. Calibrated Severity Scores (CSS) (42) are compared pre-treatment and post-treatment.

Calibrated severity scores on the ADOS-2 showed a good discrimination between clinical ASD and non-spectrum classifications (42). These calibrated severity scores are used earlier in a similar population of individuals with ASD (38, 39). For these scores, a change was computed between baseline and endpoint (43).

The Trauma Symptom Investigation Form in Autism Spectrum Disorders (TIF-ASD) (44), translated into Dutch, measures behavioral aspects of ASD related to traumatic events. The TIF-ASD assesses the impact of traumatic events on five core symptoms of autism: (1) social and (verbal) communication skills; (2) behavioral problems; (3) stereotypical & ritualistic behaviors; (4) self-care skills; (5) vegetative symptoms. The total scale consists of 20 items which are completed by parents. Items are answered on a 5-point scale ranging from never true to always. Cronbach’s alpha for the TIF-ASD was 0.92. Information on validity of the TIF-ASD is not available from the developers (44). Clinical responding on the TIF-ASD was defined as a reduction of > 25% on the total score.

The Clinical Global Impressions (CGI) scale is a well-established research rating tool applicable to all psychiatric disorders that can easily be used by the practicing clinician to meet this need.

Change in clinical global functioning was assessed at endpoint and follow-up using the Clinical Global Impression-Improvement (CGI-I) (45), rated on a 7 point scale (*very much improved-score 1- to very much worse-score 7*) by experienced child psychiatrists who were unfamiliar with the participant. Ratings were based on information about the clinical status of functioning, symptoms, and well-being in major areas of the participants life (i.e., home, school, relations). This information was provided by the coordinating therapist of the participant, who was instructed not to provide details on the treatment phase. Based on earlier research on Pivotal Response Treatment (38, 39), a clinical responder was defined as being *much improved (score 2)* or *very much improved (score 1)* on the CGI-I.

Experienced stress was measured by the Perceived Stress Scale-10 (PSS-10) (46) that assess the degree in which individuals rate their lives as unpredictable, uncontrollable, and overwhelming. The 10 self-report items were answered on a 5-point scale ranging from never applicable to very often applicable. Normscores are only available for adult participants (47), where a score above 14 is indicated as above average stress. Cronbach’s alpha for the PSS was 0.92. Convergent validity ranges from 0.56 to 0.71. (48). In the research of Hirvikoski & Blomqvist (8) 25 adults with ASD showed significantly higher scores (mean score 29, 38) on the PSS than 28 typically developing adults (mean score 19, 69). Clinical responding status was defined as a reduction of > 25% on the total score in post- treatment and/or follow up measurement compared to baseline.

#### Intervention

EMDR is a protocolized treatment (49), in which negative and adverse memories are stripped of their negative charges and become neutral in remembrance. This study used the Dutch standard EMDR protocol for children and adolescents (19).

At the beginning of every session, the participant was asked: ‘In this recent week, were there any occasions or events which gave you stress or made you upset?’ In case the participant did not come up with an event, parents were asked to assist in choosing events to focus on. With this upsetting memory, the original EMDR protocol was followed: making a stationary picture of the most upsetting moment, targeting a negative cognition which caused most distortion or stress, find out the most appropriate positive cognition, determining the Validity of Cognition (VoC, on a scale from 1 to 7)), determining the Subject Units of Disturbance (SUD, on a scale from 0 to 10) and indicating the body awareness of the tension. Then desensitization was started by offering a distracting stimulus, in which bilateral eye movements were the most frequently used distracting working memory tasks. Repeatedly the participant was asked to report about his emotions, his cognitions, his bodily awareness’s, while being distracted. When SUD decreased to zero, and the positive cognition was installed, the participant was asked: ‘Does this event reminds you of things you have experienced earlier in your life?’ If so, this earlier experience was also treated with the EMDR protocol. Reason for this question to be asked is to be sure that the past drivers of daily stress were also treated. The session ended with installing a positive competence of the participant, conform the Dutch EMDR protocol.

In the next session the therapist asked the participant if there was any SUD resting on the images of last week’s remembrance. If so, EMDR treatment was continued. If not, the participant was asked for upsetting moments over the past week.

EMDR was provided by four trained EMDR therapists. They all completed supervision of the Dutch EMDR society and were trained in extra skills for providing EMDR to children and adolescents with ASD. Supervision was given by a certified supervisor of the Dutch EMDR society^1^.

#### Procedure

After being referred to the study, a screening was done to determine if the adolescent had a PTSD diagnosis (as exclusion criteria) and met the inclusion criteria. If no PTSD diagnosis was present and all other inclusion criteria were met, the adolescent and his/her caretaker were asked for informed consent. Written informed consent was obtained from all participants and caregivers that were included in the study.

Before initiating the treatment, the ADOS and CGI-I were administered. In the four weeks prior to starting the treatment (baseline), the adolescent and one of his caregivers completed the SRS-A (adolescent and caregiver), TIF-ASD (caregiver), and PSS (adolescent) on a weekly basis. After a baseline phase of four weeks, the 10-week protocol for the EMDR treatment started, with sessions planned weekly. At each (weekly) time point, the SRS-A, TIF-ASD and PSS-10 were completed to assess change in these measures over the course of EMDR. When the EMDR was finished, the SRS-A, TIF-ASD, PSS-10, ADOS-2 and CGI-I were administered as endpoint measures. In addition, the SRS-A, TIF-ASD, PSS and CGI-I were administered at 3-month follow-up.

#### Committee of ‘experts by experience’

In order to set up the research in collaboration with the relevant population, a committee of experts by experience was conducted. This committee consisted of four former patients and a mother of one of them. All patients had a diagnosis of ASD and were treated with EMDR for their former PTSD complaints. They advised the research team on approach of participants, i.e., about formulation of patient information and the desirability of using rewards for participants. This committee was consulted before the study started, in the middle of the study and was informed of results afterward.

### Statistical analysis

Baseline descriptive statistics were obtained for all 21 participants. All available data was used and no imputation was conducted for any missing data since the explorative nature of this study. Outliers were identified by using density plots or boxplots and were adjusted to quartile (Q)1 – 1.5 * interquartile range (IQR) and Q3 + 1.5 * IQR to obtain normal distribution if necessary. Outcomes on the four measurements with the SRS-A, TIF-ASD and PSS-10 prior to start of the treatment were averaged to obtain a baseline score for each of these measures. Paired samples *t*-tests (or Wilcoxon signed ranks tests when adjusting outliers did not contribute to obtaining normal distribution) were conducted in SPSS version 25 (50) to explore changes from baseline to endpoint and from baseline to follow-up on the SRS-A, ADOS-2 (only endpoint), TIF-ASD, PSS-10 and CGI-S. Furthermore, percentages of clinical responders on the SRS-A total score and CGI-I were explored by obtaining descriptive statistics and change scores.

In addition to the baseline versus endpoint and baseline versus follow-up comparisons, linear mixed-effect models, using the lmer function of the lme4 package (51) in R (version 4.0.3; R Core Team) (52) were used to assess the change over time during the EMDR treatment on the weekly administered SRS-A total score and subscales, PSS and TIF-ASD. A per-participant random adjustment to the fixed intercept as well as the slope over time were included in the models besides the fixed effect of time. Confidence intervals (95% CI) were derived from the function *confint* in R (R Core Team) (52), with CIs that exclude zero indicating significant estimates. Also correlational analyses in SPSS were conducted to explore the relationship between (1) change in the caregiver- and self-reported total SRS-A scores over the course of EMDR and (2) participants’ age, total IQ, gender and baseline severity of ASD symptoms. A paired-samples *t*-tests was conducted to compare the baseline CSS of the ADOS-2 to the endpoint score. For all outcomes, sensitivity analyses were conducted excluding participants for which the EMDR treatment was interrupted due to the restrictions resulting from the COVID-19 pandemic (*N* = 3).

The study was approved by the Local Ethics Committee (CMO Arnhem-Nijmegen, NL60026.091.16).

## Results

### Participants

Thirty adolescents with ASD were screened for inclusion, of which 21 (12 males, 9 females) were finally included in the period from November 2017 until January 2020. Reasons for exclusion were not adhering to one of the inclusion criteria (*N* = 7) or anxiety that led to no commitment for the EMDR treatment (*N* = 2).

Table 1 shows the descriptive characteristics at baseline for the participants. On the CGI-S at baseline, the majority of participants had a score between 5 (markedly ill) and 7 (very severely ill), two participants received a score of 4 (moderately ill) and one participant received a score of 3 (mildly ill). Of the included participants, most participants received 75% of the treatment protocol.

**TABLE 1:** Baseline descriptive characteristics.

	Mean (SD)/N (%)	Range
**Gender**		
Male	12 (57.1)	
Female	9 (42.9)	
Age	15.0 (2.0)	11.9-19.3
TIQ	104.6 (9.2)	88-125
VIQ	105.4 (11.7)	85-126
PIQ	99.9 (11.4)	70-117
ADOS CSS	4.8 (2.9)	1-10
CGI - Severity	5.3 (0.9)	3-7

TIQ, total intelligence quotient; VIQ, verbal intelligence quotient; PIQ, performance intelligence quotient; ADOS CCS, autism diagnostical observation schedule calibrated severity score; CGI-Severity, clinical global impression scale - severity.

Five young adolescents were unable to complete the SRS self-report. They did not understand the questions on the report form or had severe stress while answering the questions. For them, only caregiver-reported measures were included.

Most frequent comorbidity existed of: Attention Deficit Hyperactivity Disorder (in five cases), anxiety disorders (in five cases) and depression (in six cases). Three participants had comorbid ADHD ánd depression, two participants had a comorbid depression ánd a (social) anxiety disorder. These comorbid disorders were diagnosed by well trained child psychiatrists.

From the patients that were included in the study, fourteen participants used medication on a daily basis. Among them, three used stimulants (i.e., methylphenidate, dexamfetamine, and lisdexamfetamine), four antipsychotics (i.e., risperidon and aripiprazol), two antidepressants (i.e., fluoxetine and sertraline) and five used a combination of either an antidepressant and an antipsychotic or a combination of a stimulant and an antipsychotic.

Further exploration of the data reveals that participation varies between 5 and 10 sessions. Sixteen participants (76%) completed 9 or 10 sessions of the program. One participant who received five sessions, was an “early completer,” indicating recovery from stress full events and negative mood states over last few weeks. The total response rate was therefore 81%.

### Primary outcome measure

#### Caregiver-reported social responsiveness score (SRS-A) change from baseline to endpoint and baseline to follow-up

In Table 2 the results of the paired samples *t*-tests for change in SRS scores from baseline to endpoint and baseline to follow-up are shown with means and standard deviations. For the caregiver-reported SRS-A, no significant change was found from baseline to endpoint for the total score or any of the subscale scores (all *p* > 0.05). However, a lower score at follow-up compared to baseline was found for the total caregiver-reported SRS-A score (*p* = 0.010) and for the caregiver-reported subscales Awareness (*p* = 0.002) and Communication (*p* = 0.021). No significant change from baseline to follow-up was found for the caregiver-reported subscales Social Motivation and Restricted Interests and Repetitive Behavior of the SRS-A (all *p* > 0.05).

**TABLE 2:** Means and standard deviations and results of paired samples tests.

	Baseline	Endpoint	Follow-up	Baseline to endpoint		Baseline to follow-up	
	M (SD)	M (SD)	M (SD)	*t* (df)	*p*	*t* (df)	*P*
**SRS-A caregiver-report**							
Total score	80.88 (22.82)	72.90 (19.85)	76.08 (22.17)	1.21 (9)	0.257	3.10 (11)	**0**.010**
Awareness	24.06 (6.44)	20.60 (6.11)	21.08 (7.26)	1.96 (9)	0.082	4.06 (11)	**0**.002**
Communication	25.56 (7.89)	22.70 (5.48)	22.83 (7.40)	0.88 (9)	0.401	2.69 (11)	**0**.021*
Motivation	17.51 (6.29)	16.90 (6.32)	17.58 (5.45)	0.65 (9)	0.533	1.31 (11)	0.218
Restricted Interests and Repetitive Behavior	13.75 (5.78)	12.70 (6.63)	14.58 (6.16)	0.51 (9)	0.623	1.29 (11)	0.231
**SRS-A self-report**							
Total score	70.65 (26.46)	59.63 (29.16)	61.33 (29.96)	1.15 (7)	0.286	0.86 (8)	0.416
Awareness	19.92 (8.01)	18.13 (8.79)	17.44 (9.62)	0.89 (7)	0.402	0.76 (8)	0.470
Communication	22.47 (9.43)	17.25 (10.35)	17.44 (10.21)	1.31 (7)	0.232	0.95 (8)	0.372
Motivation	15.78 (7.00)	13.00 (6.59)	14.67 (6.06)	1.16 (7)	0.283	0.39 (8)	0.705
Restricted Interests and Repetitive Behavior	12.48 (5.90)	11.25 (8.24)	11.78 (7.93)	0.23 (7)	0.825	0.62 (8)	0.553
**ADOS-2**							
Calibrated Severity Score	4.80 (2.93)	5.11 (2.87)	–	−0.74 (17)	0.470	–	–
**TIF-ASD**							
Total Score	50.39 (12.20)	50.88 (15.01)	46.75 (10.75)	0.41 (7)	0.691	3.05 (11)	**0**.011*
**PSS-10**							
Total Score	18.92 (7.00)	21.00 (8.81)	13.25 (8.42)	−1.05 (6)	0.332	2.72 (11)	**0**.020*
**CGI**							
Severity Scale	5.32 (0.65)	3.95 (1.51)	4.33 (1.30)	−3.01^a^	0.003**	−2.41^a^	**0**.016*

*p < 0.05, **p < 0.01, ^a^represents z-statistic from Wilcoxon signed ranks test; ADOS-2., autism Diagnostic Observation Schedule second edition; CGI, clinical global impression; df, degrees of freedom; M, mean; *p*, *p*-value (two-tailed); PSS-10, perceived stress scale ten item edition; SD, standard deviation; SRS-A, social responsiveness scale for adults; *t*, test statistic resulting from paired samples *t*-test; TIF-ASD, Trauma symptom investigation form in autism spectrum disorders.

#### Caregivers social responsiveness score (SRS-A) change over the course of EMDR

Mixed model analysis showed no significant main effect of time for the total caregiver-rated SRS score, Estimate = 0.87 (1.37), 95% CI: −1.84–3.75. Mixed model analyses for each of the caregiver-rated SRS subscale scores indicated no significant main effect of time for the Social Awareness (Estimate = 0.24 (0.37), 95% CI: −0.49-1.02), Social Communication (Estimate = 0.43 (0.53), 95% CI: −0.62-1.55) Social Motivation (Estimate = 0.24 (0.31), 95% CI: −0.36-0.89) or Restricted Interests and Repetitive Behavior (Estimate = 0.22 (0.27), 95% CI: −0.31-0.78) subscales. No change over the course of EMDR treatment was found in caregiver-reported Social Responsiveness.

Sensitivity analyses excluding participants of which the EMDR treatment was interrupted due to the restrictions resulting from the COVID-19 pandemic did not alter conclusions for the primary outcome measures.

### Secondary outcome measures

#### Self-reported social responsiveness score (SRS-A) change from baseline to endpoint and baseline to follow-up

On the self-reported SRS-A, no significant changes from baseline to endpoint and from baseline to follow-up were found for the total score and for any of the subscales (all *p* > 0.05). At endpoint and follow-up respectively, percentages clinical responders were 4.76 and 9.52% on the caregiver-reported SRS-A and 9.52 and 14.29% on the self-reported SRS-A.

#### Self-reported social responsiveness score (SRS-A) change over the course of EMDR

As for the caregiver-rated SRS, no significant main effect for time was found for the self-reported SRS total score in the mixed model analysis (Estimate = 0.42 (1.79), 95% CI: −3.18-4.12) and for the Social Awareness (Estimate = 0.09 (0.41), 95% CI: −0.71-0.96) Social Communication (Estimate = 0.13 (0.69), 95% CI: −1.25-1.57), Social Motivation (Estimate = 0.08 (0.48), 95% CI: −0.88-1.07) or Restricted Interests and Repetitive Behavior (Estimate = −0.04 (0.26), 95% CI: −0.56–0.51) subscales. No change over the course of EMDR treatment was found in Social Responsiveness reported by the adolescent.

There was no significant relationship between the change estimates on the caregiver- and self-reported SRS-A and participant’s age, total IQ, gender and severity of ASD symptoms. See Table 3 for correlations between mean changes in SRS-A score and participant characteristics.

**TABLE 3:** Correlations between mean change in SRS-A score and participant characteristics.

	Age	TIQ	Gender	Severity of ASD symptoms
**SRS-A**				
Caregiver-report	0.28	0.02	−0.36	0.03
Self-report	0.22	0.25	0.18	0.03

None of the correlations showed significance at α = 0.05 (two-tailed). ASD, autism spectrum disorder; SRS-A, social responsiveness scale for adults; TIQ, total intelligence quotient.

#### Severity of autism (ADOS-2)

Paired samples *t*-tests indicated no significant change from baseline to endpoint on the ADOS-2 CSS (see Table 2), indicating no change in severity of ASD symptoms after the EMDR compared to before the EMDR.

#### Trauma symptom investigation form in autism spectrum disorders (TIF-ASD)

Table 2 shows the results of the paired samples *t*-test comparing the total score on the TIF-ASD from baseline to endpoint and from baseline to follow-up. No significant change on the TIF-ASD was found from baseline to endpoint (*p* > 0.05), but a significant reduction was found from baseline to follow-up (*p* = 0.011).

Mixed model analysis showed no significant main effect of time for the TIF-ASD (Estimate = 0.98 (0.67), 95% CI: −0.34-2.40) indicating no significant change in impact of traumatic events related to ASD over the course of EMDR treatment.

#### Clinical global impression and improvement (CGI-I)

Non-parametric Wilcoxon signed rank tests on the CGI-S scores (see Table 2) showed a significant decrease from baseline to endpoint (*p* = 0.003) and from baseline to follow-up (*p* = 0.016). On the CGI-I, 52.4% of participants showed a clinical significant response at endpoint. At follow-up, 23.8% of the participants showed a significant response on the CGI-I. Chi-square analyses did not indicate a significant relationship between responder status on the CGI-I and participant’s age (11-15 y; 12-18 y), gender, IQ (below average, average, above average) or severity of ASD symptoms (low, moderate, and severe).

#### Perceived stress (PSS)

In Table 2 results are shown from the paired samples *t*-tests comparing the total scores on the PSS-10 at baseline with the total scores at endpoint and follow-up. Total score of participants is above norm score for typical adults (14) at baseline (18.92) and at endpoint (21.00). At follow up, the score is reduced to 13.25, which is below the norm score of 14. No significant change in the PSS-10 was found at endpoint compared to baseline (*p* > 0.05), but a significant reduction on the PSS-10 was found from baseline to follow-up (*p* = 0.020).

Mixed model analysis showed no significant main effect of time on the PSS, (Estimate = –0.29 (0.21), 95% CI: −0.70-0.16) indicating no significant decrease in perceived stress by adolescents over the course of EMDR treatment.

As for the primary outcome measures, sensitivity analyses excluding participants of whom the EMDR treatment was interrupted due to the restrictions resulting from the COVID-19 pandemic did not alter conclusions for any of the secondary outcome measures.

## Discussion

This explorative study is, to the best of our knowledge, the first to investigate the effectiveness of EMDR in reducing core symptoms of ASD in adolescents, diminishing experienced stress, and improving global clinical functioning. In contrast to our main hypothesis, no significant reduction of core ASD symptoms was found on the total score of the Social Responsiveness Scale (SRS) as reported by caregivers and participants, after 10 sessions EMDR on daily stress. However a significant reduction in the severity of total score of ASD symptoms and the subscales Social Awareness and Social Communication was found between baseline and follow up based on caregivers’ report. On self-reported SRS, no significant reduction on the ASD symptoms was found. Results of pre- and posttest scores of total ASD symptoms measured by the ADOS-2, showed no reduction on ASD symptoms. On the contrary, on the secondary outcome measures for self-reported stress and global clinical functioning, clear improvement is shown. Participants indicated less perceived stress from baseline to follow-up, as hypothesized. Also in line with our expectations, significant global clinical improvement was shown in 52% of adolescents on the Clinical Global Impression Improvement Scale from baseline to end-measure and 24% from baseline to follow up measure.

Findings regarding the severity of autism symptoms in this study are partly in line with the outcome of the earlier study of Lobrecht van Buuren et al. (32), as these both indicated a decrease in severity of ASD symptoms. Lobregt van Buuren et al. found significant effects on total self-report SRS-scores in adult patients with ASD and PTSD, whereas our study found no decrease in self-reported ASD symptoms, but on caregiver-reported ASD symptoms at the SRS-A, from baseline to follow up. Possibly there may be a difference in the reliability of self-reported ASD symptoms between adolescents and adults with ASD. Results from earlier studies are mixed regarding the ability of individuals with ASD to identify experienced symptoms of ASD in themselves (53–55). This may be caused by limitations in formulating thoughts about feelings and emotions, especially for adolescents with ASD (56).

In this study, effects were only found from baseline to follow-up measurement. In other research trials this tendency of delayed onset of treatment effect is called a ‘sleeper effect’, due to the time that is needed to incorporate change and gain benefits (39, 57). Discussion on whether or not a “sleeper effect” can be detected, is ongoing (58). Future research is warranted to explore the presence of a sleeper effect in (EMDR) treatment in patients with autism.

Significant global clinical improvement (52.4% was very much improved or much improved on CGI measurement) was shown in this study. This means that patients showed progress in functioning in every daylife. This might be due to stress reduction, and so led to partial decrease of ASD symptoms, and improved global clinical functioning. This finding is in line with previous research on effects of Quality of life (QoL) on treatment effect (5, 59).

Although a methodological strength of this study is the inclusion of different outcomes measures to identify treatments gains (semi-structured therapist-child interaction, caregiver-ratings, self-reports, and clinician ratings), this explorative study has also some major limitations that need to be addressed: First, due to the uncontrolled study design, the results have to be interpreted with caution, because of the possibility of interference with non-specific treatment effects. Second, this study has a small sample size, which may have introduced the risk of false negative and false positive findings.

A third limitation concerns the relatively high number of missing data, which is mainly caused by the fact that severely affected participants were included, who sometimes felt unable to carefully respond to all the weekly report forms. Also, it is important to mention that, in research literature, questions are raised about the usefulness of SRS subscales to measure treatment effects (38, 39, 60). Multiple studies using the SRS compared to other instruments, have shown that ASD symptom clusters are highly correlated (61, 62). The reliability of interpreting scores on subscale level is therefore debatable and should be done with caution (37). Furthermore, although all EMDR therapists were trained and supervised during the study, therapist adherence (as in the extension to which all therapists used the same therapeutic techniques) was not officially monitored. Lastly, no longer term follow-up assessment was performed in this study. It is therefore not possible to reflect on treatment effects on the longer term, in the light of the earlier mentioned “sleeper effect.”

In general, the results of this exploratory study provide meaningful insights, that substantiate further investigation of the application of EMDR treatment for stress reduction in adolescents with ASD.

Preferably, future studies should use a randomized control study design, but also multiple case studies designs could be of interest. It is of great importance to collect information on several follow up periods, minimize the number of missing data, monitoring therapist adherence, assessing the usability of more generic outcome measures, and looking for applicability of the treatment on a younger age. Additionally, experienced based sampling methods using modern technology (e.g., app’s) which allows almost realtime measurements of stress levels would be of interest. Also it can be valuable to collect qualitative and quantitative data about adolescents’ and parents satisfaction about method and results. This may give us more detailed information about what works in helping children with ASD to experience less stress in their daily life, and thereby hopefully increase their possibilities in social functioning.

## Conclusion

This exploratory uncontrolled study indicates that EMDR on daily stress and confusion decreases perceived stress in adolescents with ASD, and may improve their global clinical functioning (i.e., the impact of the symptoms on the patient’s ability to function). EMDR treatment has a partial and delayed effect on decreasing core symptoms of ASD.

## Data availability statement

The raw data supporting the conclusions of this article will be made available by the authors, without undue reservation.

## Ethics statement

The studies involving human participants were reviewed and approved by Medisch Ethische Commissie Oost Nederland (Radboud University Nijmegen). Research was conducted in accordance with the World Medical Association’s Declaration of Helsinki. Written informed consent to participate in this study was provided by the participants’ legal guardian/next of kin.

## Author contributions

EL wrote the first draft of the manuscript. IB-S supervised and wrote the data analyses. All authors contributed to manuscript revision, read, and approved the submitted version.

## Footnotes

1
www.emdr.nl/supervisoren ven
